# Volume Navigation with Contrast Enhanced Ultrasound and Image Fusion for Percutaneous Interventions: First Results

**DOI:** 10.1371/journal.pone.0033956

**Published:** 2012-03-20

**Authors:** Ernst Michael Jung, Chris Friedrich, Patrick Hoffstetter, Lena Marie Dendl, Frank Klebl, Ayman Agha, Phillipp Wiggermann, Christian Stroszcynski, Andreas Georg Schreyer

**Affiliations:** 1 Department of Radiology, University Medical Center Regensburg, Regensburg, Germany; 2 Interdisciplinary Ultrasound Center, University Medical Center Regensburg, Regensburg, Germany; 3 Department of Surgery, University Medical Center Regensburg, Regensburg, Germany; University of California, Berkeley, United States of America

## Abstract

**Objective:**

Assessing the feasibility and efficiency of interventions using ultrasound (US) volume navigation (V Nav) with real time needle tracking and image fusion with contrast enhanced (ce) CT, MRI or US.

**Methods:**

First an *in vitro* study on a liver phantom with CT data image fusion was performed, involving the puncture of a 10 mm lesion in a depth of 5 cm performed by 15 examiners with US guided freehand technique vs. V Nav for the purpose of time optimization. Then 23 patients underwent ultrasound-navigated biopsies or interventions using V Nav image fusion of live ultrasound with ceCT, ceMRI or CEUS, which were acquired before the intervention. A CEUS data set was acquired in all patients. Image fusion was established for CEUS and CT or CEUS and MRI using anatomical landmarks in the area of the targeted lesion. The definition of a virtual biopsy line with navigational axes targeting the lesion was achieved by the usage of sterile trocar with a magnetic sensor embedded in its distal tip employing a dedicated navigation software for real time needle tracking.

**Results:**

The *in vitro* study showed significantly less time needed for the simulated interventions in all examiners when V Nav was used (p<0.05). In the study involving patients, in all 10 biopsies of suspect lesions of the liver a histological confirmation was achieved. We also used V Nav for a breast biopsy (intraductal carcinoma), for a biopsy of the abdominal wall (metastasis of ovarial carcinoma) and for radiofrequency ablations (4 ablations). In 8 cases of inflammatory abdominal lesions 9 percutaneous drainages were successfully inserted.

**Conclusion:**

Percutaneous biopsies and drainages, even of small lesions involving complex access pathways, can be accomplished with a high success rate by using 3D real time image fusion together with real time needle tracking.

## Introduction

Currently real time image guided biopsy is performed either based on fundamental B-scan ultrasound (US) or CT fluoroscopy [1], [2]. Regular MRI and CT biopsies do not offer a real time image update. Using this approach the lesion is identified on the MRI or CT image and the tip of the biopsy needle is approximated towards the lesion while checking the position after the needle's manipulation [3], [4], [5]. The advantage of the MRI and CT based method is the excellent visualization of lesions after intravenous (iv) contrast application, which improves the perceptibility of most lesions dramatically. The disadvantage using these methods is the lack of real time image data on the needle position, which reduces the safety and pace of the procedure. CT fluoroscopy offers a real time image data update but still lacks contrast based image information because of the contrast media's dynamic. Moreover it exposes the patient as well as the physician to ionizing radiation.

A combination of contrast enhanced (ce) image data such as ceMRI, ceCT or CEUS with real time high-resolution ultrasound would be most desirable for image guided biopsies [6], [7], [8], [9], [10]. For this purpose we evaluated a recently introduced needle tracking tool, which was integrated in an ultrasound scanner (LOGIQ E9; GE Healthcare, Chalfont St. Giles, UK). For the needle tracking tool multiple three-dimensionally acquired DICOM volume data sets such as ceMRI, ceCT and CEUS can be imported and registered with real time fundamental B-scan data for volume navigation (V Nav, GE Healthcare, Chalfont St. Giles, UK) [11], [12]. Once retrieved the DICOM data set into the ultrasound scanner, the data set can be virtually rescanned and the patient anatomic landmarks can be identified with modified angulated sectional CT or MRI images of the original DICOM volume data set. The use of a trocar system with the corresponding sensor technology embedded in the needle distal dip allows the targeting of a defined marker placed beforehand in the targeted lesion. Color-coded navigational lines and the real-time display of the needle tip location with accurate markers, changing size when approaching, make it possible to perform out-of-plane approaches to the target lesion. The remaining distance to the targeted area is continuously displayed.

To our knowledge this is the first description of this advanced real time ultrasound image fusion technique, which allows the integration of previously acquired contrast enhanced DICOM image data into a real time volume navigation system (V Nav). To evaluate the general performance and efficiency of the system we first performed a phantom study with different examiners using CT based images for volume fusion. Additionally the clinical application of this system in 23 patients for different typical interventional settings is described.

## Materials and Methods

### Ethics Statement

The study was approved by our institution's ethics committee. We obtained written informed consent from our patients. Patients with contraindications for ultrasound contrast agents were excluded. Impaired kidney function was not considered as a contraindication. Because of magnetic interferences of the volume navigation system patients with pacemakers were additionally excluded.

### Volume Navigation, Image Fusion and Needle Tracking

Volume Navigation (V Nav) is achieved by placing an electromagnetic transmitter (Ascension Technology Corporation, Burlington, USA) near the area of scanning (the ‘operating volume’) and attaching a pair of electromagnetic sensors (Ascension Technology Corporation, Burlington, USA) to a bracket (CIVCO Medical Solutions, Kalona/Iowa, USA) that connects to the transducer. Both the transmitter and the sensors are connected to a position sensing unit (Ascension Technology Corporation, Burlington, USA) embedded in the ultrasound machine.

The magnetic tracking system determines the position of moveable sensors relative to a fixed transmitter within a defined operating volume. The two moveable sensors attached to the probe bracket precisely measure the magnetic field from a transmitter that is configured to generate a known set of field patterns. These transmitted patterns are arranged such that the system can resolve a unique spatial position and orientation from the values measured by each sensor. The low frequency pulsed direct current fields are unaffected by body tissues and most non-ferrous metals. The magnetic tracking system employs transient electromagnetic signal generation and processing which allows reduced sensor size, improved interference rejection, lower noise, higher update rates, and improved accuracy compared with earlier systems.

The system generates a precisely known current waveform, which is sent through a transmitter located adjacent to the operating volume. The transmitter converts this current into a magnetic field, which is detected by a sensor and outputs as a small voltage. This tiny signal is then amplified and digitized.

The data stream is processed by a field programmable gate array and two floating point digital signal processors that analyze the amplitude, the shape and the frequency content of the data stream. Mathematical corrections are applied to remove conductive metal effects, external noise, and other errors. The values are then fed into an algorithm that determines the position and orientation of the sensor relative to the transmitter.

With this technique, the accurate position and orientation of the Ultrasound probe within the tree-dimensional operating volume around the V Nav transmitter can be determined precisely. Volume Navigation also enables the use of so called ‘GPS Markers’. A GPS Marker is a crosshairs cursor that can be placed within the live Ultrasound image in order to mark a point of special interest. As soon as the probe is moved during scanning, the crosshairs cursor changes to a square which becomes larger the more the probe is moved away from its original scanning position. Vice versa, the square-shaped cursor becomes smaller when the probe gets closer to the original position and changes to crosshairs once the marked point of interest is accurately scanned again.

Utilizing the three-dimensional position and orientation data of the ultrasound probe, the ultrasound image can be fused with spatial data sets from CT or MRI.

For V Nav Image Fusion, the DICOM volume data set from pre-acquired CT or MRI exam is loaded into the ultrasound system. By manually registering the live ultrasound image to the according image area of the CT/MRI data, both images can be synchronized. Registration is either done by defining a common plane plus one additional, common point or by defining at least three common points. Once synchronized, the live ultrasound image and the corresponding CT/MRI image section are displayed either side-by-side or can be overlaid.

These different modalities complement each other excellently, as lacking information from one modality can be provided by the other modality in terms of spatial, contrast or temporal resolution. Due to point-to-point image fusion of volume data from CT, MRI and US examinations, even lesions hard to detect in US can be reached during US-guided interventions.

For V Nav Needle Tracking, an additional, small V Nav sensor is positioned in the distal tip of a sterile, coaxial needle, thus locating the exact position of the needle tip and projecting the needle's path during interventions.

By knowing the exact location and direction of the needle in relation to the ultrasound probe and therefore, to the ultrasound image itself, the projected needle path and actual needle tip position can be represented graphically on the real-time ultrasound image. This augmented reality view enables interventional procedures to be planned using any needle insertion path, including an approach that is out of plane with the ultrasound image. As the needle is inserted, the needle path and trajectory are projected onto the current image. Because the sensor is at the distal tip of the needle, the tip and trajectory graphics are correct even when the needle bends. Combining V Nav Needle Tracking with Image Fusion, the needle tip position and its projected path may also be overlaid on the fused CT/MRI image.

The eTRAX needle tracking system (CIVCO Medical Solutions, Kalona/Iowa, USA) is a sterile, disposable two-part coaxial needle consisting of an 16G sheath (trocar) and an 18G stylet (needle). A reusable 0.9 mm sensor is embedded in the distal tip of the hollow stylet. Once the two-part coaxial needle has been placed at the anatomy of interest, the stylet with the embedded sensor is removed and an 18G or smaller instrument can be placed through the sheath (trocar) to reach the anatomy of interest.

A GPS marker may be used to accurately mark the position of the needle tip before the stylet and therefore, the sensor is removed. This marker helps to relocate the targeted area if the probe has been moved or if the targeted area is no longer clearly visible in ultrasound.

### *In vitro* study

**Figure 1:**
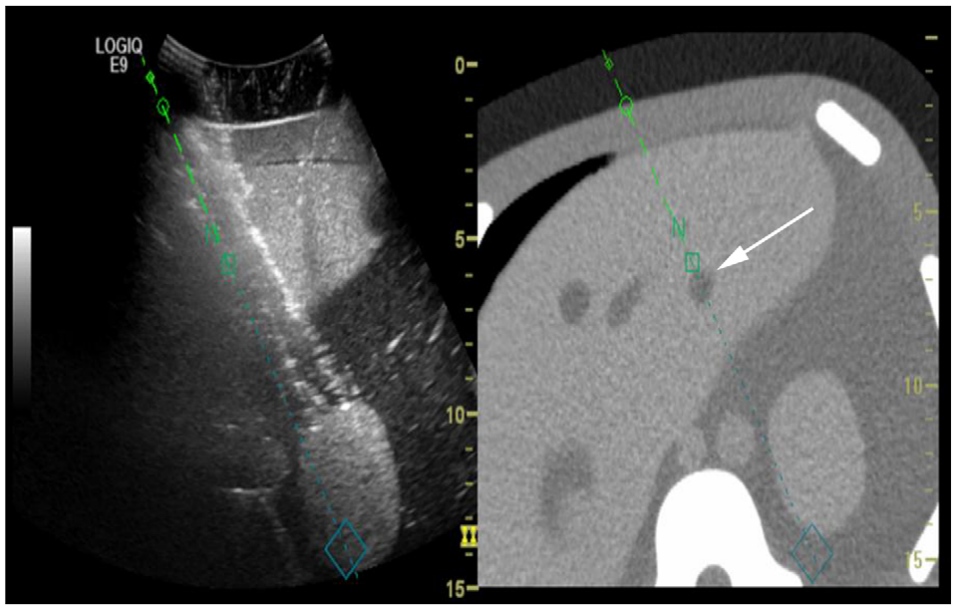
Image fusion of the CT and B-Scan of the liver phantom used in our preclinical trial. Puncture of a simulated small lesion (white arrow) in the left liver lobe. The lesion is not visible on the ultrasound scan. The lefts part oft the figure shows the real time ultrasound image, the right side the registered CT scan.

First, we performed an in vitro study to analyze the time efficiency and success of simulated puncture employing the V Nav needle tracking system. Using a liver phantom with corresponding CT images for image fusion, a lesion with a diameter of 1 cm, which was located 5 cm below the surface, was independently punctured by 15 different physicians both in standard US guided free hand technique and employing the V Nav technique (Figure 1).

When employing the navigation system, real time US images were synchronized with CT images using plane and point registration to achieve optimal fusion overlay of the target lesion. Then the time necessary to reach the lesion (location of the 18G biopsy needle documented in the center of the lesion) was recorded. An independent reader evaluated the digitally archived cine-loops, considering success in reaching the lesion and the time necessary to achieve the whole task.

Statistical analysis was performed using the Wilcoxon Test and Pearson's correlation analysis with SPSS for Windows (Version 16.0.1, SPSS Inc, Chicago, USA).

### Clinical Trial

We performed an US-guided diagnostic biopsy in 12 patients and US-guided interventions (9 drainages in 8 patients and 4 radiofrequency ablations (RFA) in 3 patients) by using V Nav image fusion with ceCT (18 cases) or ceMRI (5 cases). For patient characteristics, see Tab. 1.

**Table 1:** Patient characteristics.

Patient No.	Age at time of intervention	Sex	Intervention	Diagnosis	Duration (min)
1	41	female	Biopsy Liver	Metastasis of Neuroendocrinous Tumor	23
2	74	male	Biopsy Liver	Metastasis of Melanoma	19
3	68	male	Biopsy Liver	Metastasis of Pancreatic Carcinoma	18
4	47	male	Drainage	Pelvic Abscess	44
5	50	male	Biopsy Liver	Focal Fibrosis	12
6	48	female	Biopsy Breast	Intraductal Carcinoma	19
7	70	male	Biopsy Liver	Metastasis of Colorectal Carcinoma	15
8	71	male	Biopsy Liver	Metastasis of Stomach Carcinoma	13
9	18	female	Drainage	Abscess due to Traumatic Pancreatitis	42
10	52	male	Biopsy Liver	Hepatocellular Carcinoma	17
11	49	female	Biopsy Liver	Cholangiocellular Carcinoma	12
12	76	male	Biopsy Liver	Focal Inflammation of the Liver associated with Xanthogranulomatous Pyelonephritits	18
13	53	male	Drainage	Abscess after partial Liver Resection	28
14	70	male	RITA	Hepatocellular Carcinoma	54
15	69	female	Biopsy Liver	Metastasis of Thymoma	11
16	66	male	Drainage	Abscess after Rectum Resection	23
17	67	female	Biopsy Inner Abdominal Wall	Metastasis of Ovarial Carcinoma	15
18	59	male	RITA	Liver Metasatasis of CRC	48
19	45	male	Drainage	Abscess after partial Stomach Resection	25
20	71	male	Drainage	Seroma after Split Liver Transplantation	23
21	58	male	RITA 2×	Liver Metastases of Coloractal Carcinoma	68
22	59	female	Drainage	Abscess after Liver Transplantation	31
23	76	female	Drainage 2×	Abscesses due to Pancreatitis	37

For all 23 patients additionally CEUS was performed by an experienced US specialist (more than 5000 US examinations). We employed a LOGIQ E9 ultrasound scanner (GE Healthcare, Chalfont St. Giles, UK). First, a basic scan of the relevant area was carried out in sweep technique, using a multi frequency probe (C1-5-D, 1–5 MHz, LOGIQ E9). To assess the vascular patterns, we added color coded duplex sonography and power Doppler scans of the examined area.

Afterwards, a CT and/or MRI DICOM volume data set was retrieved into the LOGIQ E9 V Nav. Image fused real time ultrasound with ceCT or ceMRI was achieved by plane locking adapted axial US slices with the axial slices of the other modalities. Using the overlay technique and three point registration, we established an excellent point to point accuracy in the B-scan. Then additionally performed CEUS was acquired for better identification of the tumor lesions or abscess formations, which were the targets of the intervention or biopsy.

For CEUS a bolus of 2.4 ml second-generation contrast agent (SonoVue®, Bracco Imaging, Milan, Italy) was injected in a peripheral cubital vein followed by a 10 ml saline flush.

The indication for the biopsies, drainages and radiofrequency ablations (RFA) was established interdisciplinary together with the patients' primary physicians. We administered local anesthesia and, when drainages were required, systemic analgesics as well, while monitoring cardiac and respiratory parameters.

For the interventions, a GPS marker was placed in the target lesions in the fused images from CEUS and CT or MRT. Using a sterile eTRAX Needle Tracking system with an embedded sensor in the distal tip together with the Needle tracking software, a virtual pathway to the lesions was calculated. The markers used for calculating the pathway were region-dependent, changing their color and the size of a virtual rectangle from brown (above) or red (below) to green when approaching the ideal calculated line. Additionally the size of the rectangle became smaller when reducing the distance to the target lesion (Movie S1). Thus, the marker representing the needle tip on the skin before the beginning of the intervention was shown as a brown or red rectangle, whereas the needle tip in the center of the targeted lesion was depicted as a green dot. Digital cine-sequences with a minimum duration of 60 seconds were recorded for documentation purposes.

To assess the time necessary for performing an intervention either under CT control or US-guided using V Nav, we randomly chose ten biopsies and ten drainages under CT control and compared those values to the time needed for V Nav US-guided procedures.

Time was measured beginning from the moment the patient was ready for the intervention - positioned on the examination table, all necessary preparative measures taken - to the moment the patient was able to leave the examination table.

## Results

### *In vitro* study

For the preclinical study on a liver phantom applying CT image fusion with the V Nav needle tracking system 15 examiners with different ultrasound experience performed the puncture of the simulated lesion successfully. Details on the preclinical phantom study are summarized in table 1.

The average time needed for successful puncture of the simulated lesion in conventional ultrasound guided freehand technique without V Nav was 116 seconds (range 46 to 342 seconds). All examiners needed significantly less time to perform a successful puncture with the aid of V Nav, the average time here being 50 seconds (range 15 to 128 seconds, p<0.05). In 6 of 15 examiners the difference between V Nav guided lesion access and freehand access was less than 30 seconds. In 9 of 15 cases we found a major difference between 50 and 214 seconds between the different approaches. The mean difference between freehand and V Nav guided lesion access was 66 seconds.

### Clinical Trial

All 10 diagnostic liver punctures were technically successful. The punctured lesions had a mean diameter of 11 mm (range 7–15 mm). Histology revealed metastases in 6 cases, hepatocellular carcinoma in one case, cholangiocellular carcinoma in another case and a single case of focal inflammation of the liver associated with xanthogranulomatous pyelonephritis as well as one case of focal liver fibrosis leading to reduced liver synthesis. The punctures were performed using an oblique subcostal access-way. Five lesions were located in the liver segment VIII, three lesions in segment II and 2 lesions in segment IVb. The extension of the lesion was assessed using CEUS. Based on the virtual guidance line a trocar with an electromagnetic sensor was positioned at the rim of the lesion. The center of the lesion as seen in CEUS was aligned with the center of the lesion assessed by sectional imaging (ceCT or ceMRI). Using an 18 G hollow puncture needle inserted through the trocar, two samples were acquired for each lesion. The sole complication in these biopsies was a hematoma within the liver capsule in a patient with reduced coagulation due to liver cirrhosis, which was resolved after 6 days.

**Figure 2:**
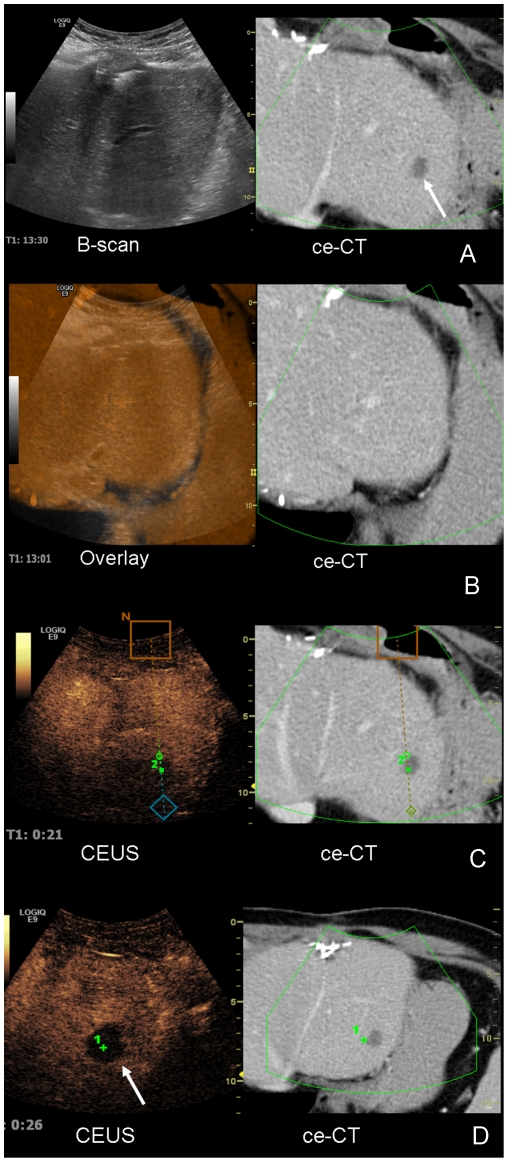
Image fusion (ultrasound and CT) for interventional planning for local radiofrequency ablation. A 67 years old patient with colorectal carcinoma and several partial liver resections in his history showed a new solitary liver metastasis in segment II of the liver, clearly visible in ceCT. The referring surgeons requested a local radiofrequency ablation of the metastasis. Figure 2 A. The metastasis cannot be detected in fundamental B-scan, but in the ceCT on the right side. Figure 2 B. For image fusion the contrast enhanced CT scan is color-coded and superimposed onto the fundamental B-scan. Figure 2 C. CEUS clearly shows the metastasis, and is therefore used for planning of the radiofrequency ablation. Figure 2 D. CEUS control after radiofrequency ablation with point registration shows complete necrosis in the area of the former metastasis with a safety margin of over 1 cm in all directions.

RFA of 4 liver lesions was performed in 3 patients (1× hepatocellular carcinoma, 3 liver metastases from colorectal carcinoma). The lesions were not reachable by an in-plane CT access. Therefore we performed an optimized angulated approach for the RFA probe using a trocar with an electromagnetic sensor for virtual guidance lines placed in the ablation's target lesion successfully in all 4 lesions (figure 2).

**Figure 3:**
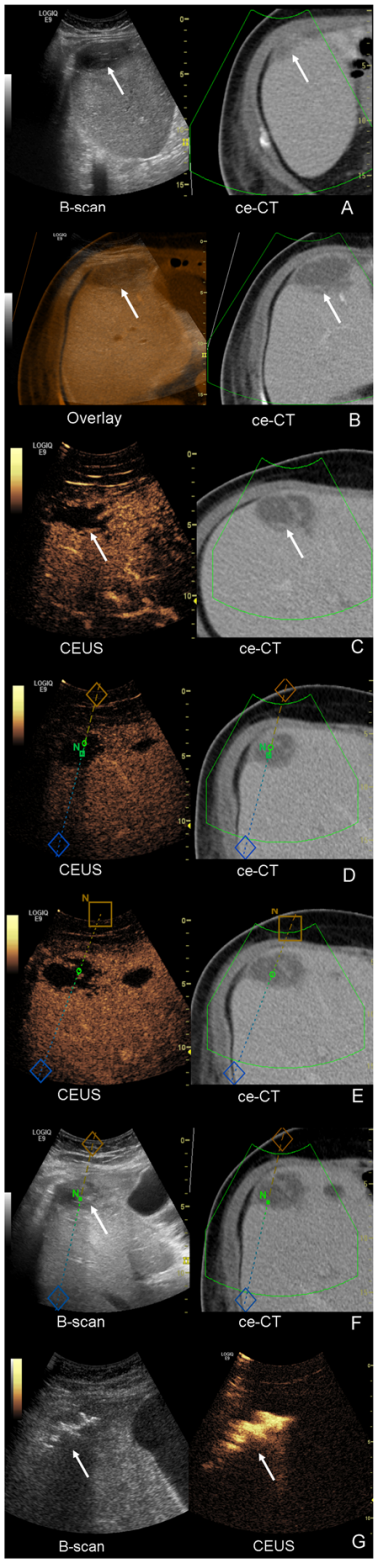
Abscess drainage performed with image fusion (ultrasound and CT). A 45 years old patient presented with elevated infection parameters and fever after partial resection of the stomach. Contrast-enhanced CT showed an abscess. The referring surgeons requested a drainage. Figure 3A. Fusion of fundamental B-Scan and contrast-enhanced CT for planning the intervention shows subcapsulary a mostly liquid formation in liver segment VIII (white arrow). Figure 3B. Overlay technique (color-coded CT and fundamental B-Scan) for optimized fusion. Figure 3C. CEUS shows a clearly demarcated mostly liquid formation in liver segment VIII and excellent image fusion with ceCT. Figure 3D. Planning of needle tracking (V Nav). The virtual puncture line is showing the anticipated end point of the needle in the middle of the formation (green circle). The subcostal cutaneous beginning of the puncture line lies outside the displayed plane (brown square marked ‘N’). Figure 3E. The needle tip now lies in the center of the formation (small green quadrant marked ‘N’) and next to the planned location (green circle). Figure 3F. B-scan control confirms needle tip within the formation (white arrow). Figure 3G. Drainage has been inserted in Seldinger technique. US contrast agent applied over drainage is documented within the abscess formation in parallel imaging (B-scan and contrast mode).

The percutaneous abdominal abscess drainages (six patients with an intraperitoneal and two patients with an extraperitoneal abscess) were successful in all 8 patients. We placed the eTRAX trocar into the abscess formations based on a virtual guidance line and then inserted the drainages (drainage size between 10 G and 14 G) using Seldinger technique. The image fusion allowed a better identification of bowel loops surrounding the abscesses. Two patients had an abscess due to traumatic or acute pancreatitis. Two patients had an abscess or seroma after liver transplantation. One patient developed abscesses after partial liver resection, after rectum resection and after partial stomach resection, respectively. Another patient developed a pelvic abscess as a complication following an ileocecal resection due to Crohn's disease (Figure 3). The visualization of the pleural recess was a huge advantage for the obliquely oriented access. Having a real time image fusion with CEUS and ceCT or ceMRI, the hyperperfused abscess walls as well as vessels en route to the abscess were visualized clearly.

We additionally used the V Nav technique for a biopsy of a breast tumor detected by ceMRI. This lesion proved to be an intraductal carcinoma.

As far as the time needed for the US-guided interventions is concerned, we experienced a clear learning curve resulting in time spans similar to and sometimes even shorter than those needed in CT-guided interventions. The average time needed for an US-guided biopsy was 16 min (range, 11–23 min), whereas for drainages we needed an average time of 32 min (range, 23–44 min).

To achieve an adequate comparison to CT-guided interventions, we randomly selected 10 CT-guided biopsies and 10 CT-guided drainages from our hospital as a comparison. The resulting values for the CT-guided procedures were 14 min (mean, range: 9–19 min) for biopsies and 41 min (mean, range 27–51 min) for drainages.

## Discussion

Conventional ultrasound is a widely available imaging modality, which allows real time imaging without any ionizing radiation. Because of these qualities it is a perfect modality to guide interventional procedures such as diagnostic biopsies and therapeutic drainages. Compared to other radiological imaging modalities such as ceCT or ceMRI fundamental ultrasound still has a certain disadvantage in detecting lesions within organs. For example, isoechogenic tumor lesions, particularly those measuring less than 10 mm, are difficult to detect [7], [13]. This especially applies to liver lesions in inhomogeneous liver parenchyma, e.g. in cirrhotic livers, where a hepatocellular carcinoma is a highly relevant finding. Using ultrasound the detection of liver lesions can be improved by using CEUS [8]. Recent studies showed that CEUS permits a high diagnostic specificity and sensitivity, comparable to these parameters in CT [7], [8], [10], [12], [14], [15], [16], [17], [18]. MRI may offer additional advantages due to the use of liver-specific contrast media and diffusion-weighted images, permitting the detection of small tumors [13], [19]. Therefore even experienced examiners cannot completely counterbalance the method's limitations. Still, in a regular setting the dynamic contrast enhanced ultrasound images cannot be superimposed onto the fundamental ultrasound to allow an interventional guidance.

For a definite characterization of these small lesions, a biopsy is often required. Image fusion of CT or MRI examinations with CEUS often permits an US-guided diagnostic biopsy of the relevant lesions to achieve diagnostic security and thus contribute valuable information influencing the following treatment.

In 2009, our group described the first application of real time image fusion for the diagnostic evaluation and characterization of liver tumors in 20 patients [12]. Now we augment this image fusion method by evaluating a 3D volume navigation system, which uses a magnetic sensor in the tip of the distal trocar for real time interventional guidance In this study we describe an integrated ultrasound and navigation system, which allows the fusion of 3D DICOM data sets based on ceCT, ceMRI or CEUS imaging with high resolution real time ultrasound to allow real time guidance for interventions. Thus the described V Nav system allows the combination of a radiation free real time imaging modality based on ultrasound with excellent diagnostic imaging methods such as ceCT, ceMRI and CEUS. Because of the real time character of ultrasound a safe approach for biopsies and other interventions can be achieved by avoiding blood vessels and generally reducing complications by displaying the actual pathway of the biopsy within the volume data together with the hazards this pathway may contain. Thus the least risky approach for securely reaching the target lesion can be determined with image fusion before the interventions involving complex, angulated access ex-plane ways. This theoretically allows complex lesion approaches next to the pulmonary recess or in a close neighborhood of the heart or large arterial vessels. Phantom studies showed already the markedly improvement for puncture accuracy applying 3D and 4D ultrasound [20]. The benefit of contrast enhanced ultrasound for the biopsy of musculoskeletal masses without the implementation of 3D navigation was recently published by another group [6].

Ultrasound-guided biopsies or interventions lack any ionizing radiation exposure, whereas complex CT-guided biopsies and interventions often make the use of fluoroscopy necessary, which goes hand in hand with a high radiation exposure for both the patient and the performing physician. According to our data, V Nav is equally fast compared to CT when the time necessary for the procedure is concerned. Particularly in drainage placement, US-guided interventions may prove to be more time-efficient, as the frequent control scans which are routine in CT-guided drainages and the associated delay are no longer necessary.

Limitations of this study are, on the one hand, the small number of patients, the lack of homogeneity of the patient population and the lack of control group undergoing conventional, CT- or US-guided interventions. We planned this project as a pilot study examining the feasibility and possible advantages and disadvantages of ultrasound-guided interventions with V Nav image fusion. Follow-up studies with larger patient collectives are necessary to conclusively rate the value of punctures under V Nav with Needle Tracking and CEUS guidance.

The combination of real time 3D navigation employing a magnetic sensor within the trocar (V Nav system) and image fusion can be helpful in performing punctures with complex access way and also may permit a reduction of the radiation dose the patients are exposed to, when compared to patients undergoing CT-guided interventions or even interventions using fluoroscopy, while at the same time delivering real-time images of the procedure.

We expect that the technique used in this study may enable less experienced examiners to successfully perform complex biopsies or place drainages involving complex access ways.

## Supporting Information

Movie S1 **Image fusion guided approach to a subcapsular liver lesion.** The markers used for calculating the pathway is depicted in real time region-dependent, changing their color and the size of a virtual rectangle from brown (above) or red (below) to green when approaching the ideal calculated line. Additionally the size of the rectangle becomes smaller when reducing the distance to the target lesion. Thus, the marker represents the needle tip on the skin before the beginning of the intervention is displayed as a brown rectangle, whereas the needle tip in the center of the targeted lesion is depicted as a green dot.
(MPG)
